# Influence of the drying mode of support on the properties of Pd/Al_2_O_3_–ZrO_2_ materials used for methane combustion

**DOI:** 10.1038/s41598-023-47630-7

**Published:** 2023-11-20

**Authors:** C. Amairia, S. Fessi, M. Mhamdi, A. Ghorbel, J. Llorca

**Affiliations:** 1 Laboratoire de Chimie des Matériaux et Catalyse, Département de Chimie, Faculté des Sciences de Tunis, Université Tunis-El Manar, Campus Universitaire, 2092 Tunis, Tunisia; 2 Chemistry Department, College of Science, Al Baha University, Al Bahah, 65779, Saudi Arabia; 3 Laboratory for the Application of Materials to the Environment, Water and Energy LAMEEE, Faculty of Sciences Gafsa, University of Gafsa, 2112 Gafsa, Tunisia; 4 Chemistry Department, College of Science and Arts Al Makhwah, Al Baha University, Al Bahah, Saudi Arabia; 5 Universitat Politècnica de Catalunya, Barcelone, Spain

**Keywords:** Environmental sciences, Chemistry, Materials science

## Abstract

This work constitutes a new trial to enhance the properties of palladium supported on alumina modified with zirconium used as catalysts for methane combustion. The effect of the support drying mode is studied. For this aim, Al_2_O_3_-ZrO_2_ binary oxides with zirconium loading of 2 and 5% in weight were prepared using sol–gel process then dried under ordinary or supercritical conditions. Palladium with a loading of 0.5% was deposited on the support by wet impregnation. Several techniques have been used to investigate differences between the two types of the derived catalysts.

## Introduction

Natural gas provides an attractive alternative to gasoline as an economic fuel source since it is an abundant supply and it is readily available for decades^1^. Also, the use of natural gas has environmental benefits because its ignition temperature for combustion is lower than that of gasoline. Natural gas does, however, consist primarily of methane, which is a potent greenhouse gas^2^. Thus, motivation exists to develop ways to remove it by complete oxidation. Compared to conventional thermal combustion, catalytic combustion is an environmentally friendly technology for power generation. It offers the possibility to product heat and energy at low temperatures and to reduce the emissions of pollutants^3–6^. A majority of the investigations directed towards combustion has involved noble metals^7^. Only Pd and Pt have been widely explored for lower alkane combustion, due to the higher volatility of the other noble metals^7^. For methane combustion, there is a general consensus that palladium-based catalysts are more active whereas platinum group metals are preferred for the combustion of higher hydrocarbons^8^. The literature contains strong evidence that the catalytic properties of Pd-based systems greatly depend on the nature of the support^9–13^. In previous papers^14–17^, we have successfully stabilized and improved the catalytic activity of palladium supported on alumina by introducing zirconia. The aim of the present work is to enhance the textural properties of Pd/Al_2_O_3_–ZrO_2_ materials which may lead to better dispersion of the active species known as a key factor for the catalytic activity of such materials. According to Pajonk^18^, when porous textures and very high surface areas are preferred, it is required to preserve the textural properties of the wet gel by adopting appropriate drying methods. Among these methods, the supercritical drying process as well as the freeze-drying method, are particularly suited as described earlier^19,20^. In this work, we have adopted the former which consists on drying the wet gel on an autoclave at a temperature above the critical temperature of solvents^19^. There are already, works in literature, that accounts of aerogels as supports of catalysts or as catalysts themselves^21–26^. Some trials have been done specifically to prepare single oxides such as alumina^27–29^ and zirconia^30–32^. Komarneni et al.^33^ have prepared SiO_2_–Al_2_O_3_ aerogels; others have prepared TiO_2_-SiO_2_ aerogels^34,35^. For us, we are interested in the preparation of Al_2_O_3_–ZrO_2_ aerogels and in their comparison with xerogels when used as supports for Pd-based materials destined to methane combustion.

## Experimental section

### Synthesis procedure

According to previous work^17^, combination of sol–gel chemistry with conventional wet impregnation led to more active catalysts. Furthermore, the use of zirconium acetylacetonate Zr(C_5_H_7_O_2_)_4_ as zirconium precursor enhances the properties of Pd/Al_2_O_3_–ZrO_2_ materials^16^. Thus, these two preparation parameters were maintained during the synthesis of the samples studied in this paper.

#### Support preparation

As already described^17^, a mixture of Al-sec butoxide and sec-butanol with an alkoxide concentration of 1 M is stirred until the total dissolution of the aluminum precursor. Zirconium acetylacetonate is then added to have a weight loading of 2 and 5% of Zr. Hydrolysis is assured by the addition of acetic acid with a molar ratio $\frac{{n}_{C{H}_{3}COOH}}{{n}_{alkoxide}}=4$. The obtained gels are divided into two parts. The first one is dried in an oven at 70 °C for 24h to obtain xerogels. The second one is introduced in an autoclave to be dried under supercritical conditions of the solvent to have aerogels.

#### Catalyst preparation

A weighed amount of palladium acetylacetonate is dissolved in acetone then slowly added to the support by impregnation. All prepared catalysts contain a Pd weight loading of 0.5%. After being dried in oven at 70 °C for 24h, these materials were calcined at 500 °C under oxygen flow for 2 h. Hereafter, catalysts are referred as Imp-Xy and Imp-Ay with:

X: support xerogel; A: support aerogel; y: zirconium loading.

#### Characterization techniques

Specific surface area measurements were carried out through N_2_ adsorption on an ASAP 2020 using the BET method. Samples were previously degassed for 4h at 200 °C under vacuum. X-ray diffraction measurements were carried out on an automatic diffractometer (Philips Panalytical). The Cu Kα radiation (λ = 1.5458 Å) and the following experimental conditions were used: 2θ range: 10–70°, step size = 0.02° and time per step = 4.80 s. Pd dispersion was determined by the dynamic pulsed hydrogen chemisorption technique using an Auto Chem II Apparatus. Prior to measurements, the sample was reduced in 5% H_2_/Ar flow at 300 °C. PdO thermal stability was studied using temperature programmed oxidation. 50 mg of the sample was loaded into a quartz reactor and reduced in a 5% H_2_/Ar flow, then heated in a 5% O_2_/He flow (30 mL min^−1^) at a temperature rate of 10 °C min^−1^ from room temperature to 1000 °C. The samples were cooled to 300 °C under the same flow. Pd reducibility was determined by using a stream of 5% H_2_/Ar at a flow rate of 30 mL min^−1^. Prior to measurements, 50 mg of the sample was heated for 1 h to 500 °C under 5% O_2_/He stream. Crystallinity, particle size and dispersion of Pd were investigated by TEM using a Philips G20 Ultra-Twin instrument operating at 100 kV. The powder was ultrasonically dispersed in ethanol and the suspension was deposited on a copper grid coated with a porous carbon film. X-ray photoelectron spectroscopy (XPS) was also used to characterize these samples via a SPECS system equipped with an XR50 source operating at 250 W and a Phoibos 150 MCD–9 detector. The pass energy of the high–resolution spectra was set at 0.1 eV. Pd catalytic properties were evaluated in methane combustion under 1% CH_4_/4% O_2_ and balance Helium stream with a total flow rate of 6 L h^−1^. The catalytic tests were performed at atmospheric pressure in a dynamic microreactor over the oxidized sample (100 mg). The effluent was analysed on-line with a gas chromatograph equipped with a thermal conductivity detector (TCD) and a Porapack column.

### Results and discussion

#### Catalysts texture

Table 1 lists data from the nitrogen adsorption measurements. As can be seen, when palladium is supported on aerogels, the specific surface area of the derived catalysts is significantly higher independently of the zirconium content. This result corroborates the excellent thermal stability of aerogels in contrast of xerogels and evidences the influence of the drying mode^36^. On the contrary, the increase of the Zr content in the catalyst is accompanied by a decrease in the specific surface area and this is more pronounced for aerogels (Sample Imp-A5). This result is understandable since the sample Imp-A5 is characterized by bigger pore diameter compared to Imp-A2, Imp-X2 and Imp-X5.

**Table 1:** BET surface area, pore diameter and pore volume of Imp-Xy and Imp-Ay catalysts.

Catalyst	S_BET_ (m^2^/g)	D_p_ (nm)	V_p_ (nm)
Imp—X2	210	8.9	0.57
Imp—X5	218	9.1	0.54
Imp—A2	333	7.6	0.67
Imp—A5	243	23.2	1.23

**Figure 1:**
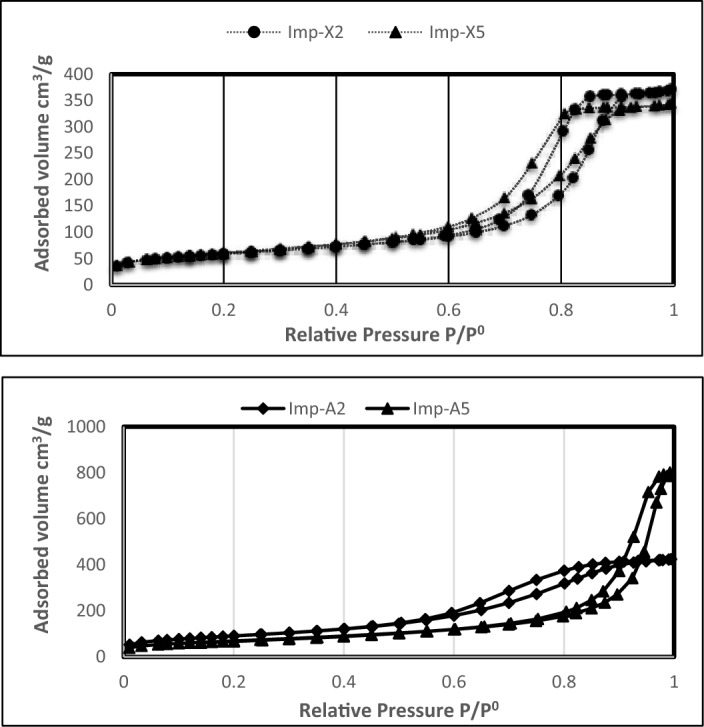
Influence of the drying mode and the Zr content on the N_2_ adsorption isotherms.

The examination of the N_2_ adsorption isotherm of both aerogels and xerogels catalysts (See Fig. 1) shows hysteresis cycles indicating that all samples are mesoporous. However, the type of the hysteresis seems to be affected by the drying mode suggesting a modification in the pore geometry.

**Figure 2:**
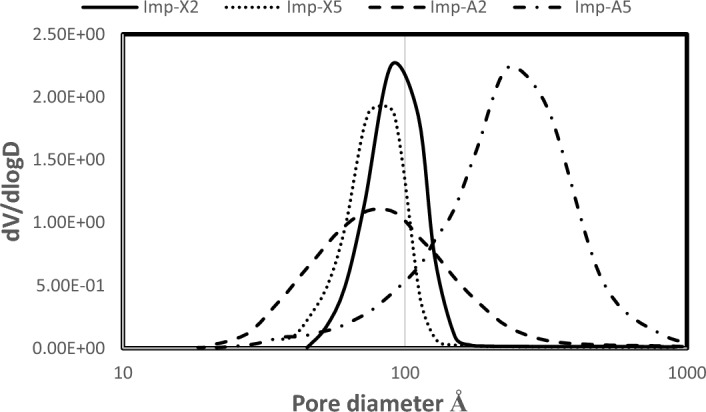
BJH pore distribution of the prepared catalysts calcined at 500 °C.

Figure 2 exhibits the BJH pore distributions of all catalysts. Similarly to results observed in a previous work^17^, the use of zirconium acetylacetonate leads to homogeneous pore distribution. In fact, only one type of pores is obtained for all catalysts. However, when comparing aerogels to xerogels, we can note a wider pore distribution in aerogel samples. Similar results were obtained by S. Alwin et al.^37^. In fact, these authors were interested in the preparation of aerogels TiO_2,_ and they have found that the pore size distribution is very wide for some samples with the average pore size 23 nm. C. Erkey and M. Türk^38^ have also reported the narrow pore distribution in aerogels. By comparing the pore size in all samples, Imp-A5 is seen to exhibit the highest pore diameter. This could be explained by the fact that aerogels in general could have smaller or bigger pores as it has been advanced by G. Horvat et al.^39^ and may be the sample Imp-A5 and its zirconium content and what happens during its preparation together with the removal of the solvent under supercritical conditions, are the possible causes of the big pores and the moderate specific surface area observed for this catalyst. Based on a work done by A. Taavoni-Gilan et al.^40^ interested in the investigation of the zirconium content effect on the properties of Al_2_O_3_–ZrO_2_ system prepared via sol–gel method, they have noted a decrease in the specific surface area of these materials accompanied by an increase of the pore size for high zirconium loadings. And according to them, this phenomena is due to the decrease in the γ-alumina content. This hypothesis could be accepted in our case, and it will be demonstrated later in the XRD results section. Also, as advanced by Z. Tian et al.^41^, during the drying of the gel, the capillary forces, originated by the surface tension of the liquid phase, tend to densify the solid by closely packing together the elementary particles, destroying the most fragile part of the three-dimensional network and reducing the porous volume of the resulting solid. However, with supercritical drying, the gel collapse will be suppressed due to disappearance of the capillary pressure. The fibroid structure of the gel will be maintained, and large pores will be present in the aerogel after drying.

#### Catalysts crystallinity

**Figure 3:**
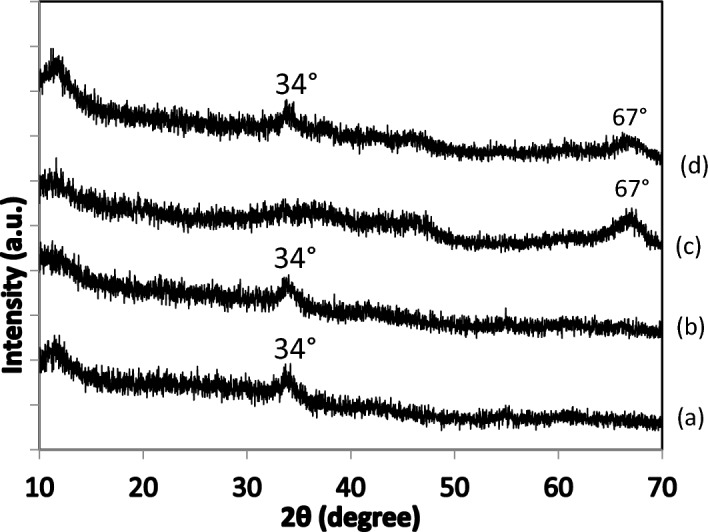
XRD patterns of the prepared catalysts calcined at 500 °C: (**a**) Imp-X2, (**b**) Imp-X5, (**c**) Imp-A2 and (**d**) Imp-A5.

XRD analysis of all samples (See Fig. 3) shows no diffraction peaks relative to zirconia phases which is in accordance with literature, where these phases have been observed only for Zr contents higher than 5% wt.^15^. No alumina phase was detected for catalysts derived from xerogels whereas a broad peak at about 67° characteristic of γ alumina^42^ was observed in Imp-Ay samples, similarly to the results obtained by Ponthieu et al.^43^. In fact, they showed that when alumina aerogel is prepared from aluminum-sec-butoxide and sec-butanol, γ alumina is detected in the temperature range 400–1150 °C. By comparing the width of this peak in both samples (Imp-A2 and Imp-A5), we can note that it is larger in Imp-A2 sample. We can thus conclude that γ alumina fraction in Imp-A5 is lower which is in accordance with the hypothesis advanced for the explanation of the larger pores observed for this sample. Another broad peak at about 34° was observed on samples derived from xerogels and on Imp-A5. This peak could be attributed to the (101) crystallographic plane of tetragonal PdO^42^. The absence of such peak in the XRD pattern of Imp-A2 and its low intensity on Imp-A5 suggest that drying the support under supercritical conditions leads to better dispersed Pd particles.

### Palladium dispersion

Table 2 lists Pd dispersion (D %), Pd particle size and the metallic surface area of all samples. In comparison with previous results obtained on similar catalysts prepared by using the same preparation route but with zirconium butoxide as precursor^17^, better palladium dispersion is obtained when zirconium acetylacetonate is used. Also, when comparing catalysts with 2 and 5% in weight of Zr, better dispersion of Pd is noted when the zirconium content is higher independently of the drying mode. This observation is in good accordance with our previous work when we have noted the improvement of dispersion with zirconium loading. By referring to the aerogels samples results, palladium seems to be more dispersed as expected from the XRD results. The Pd particle size is larger in the xerogel catalysts with respect to aerogels, which results in lower exposed metallic surface area.

**Table 2:** Pd dispersion (%D), Pd particle size and metallic surface areas of Imp-Xy and Imp-Ay.

Catalyst	D (%)	Pd particle size (nm)	Metallic surface area (m^2^/g sample)
Imp—X2	31	3.7	0.68
Imp—X5	45	2.5	1.01
Imp—A2	42	2.7	0.94
Imp—A5	49	2.3	1.09

#### PdO thermal stability

**Figure 4:**
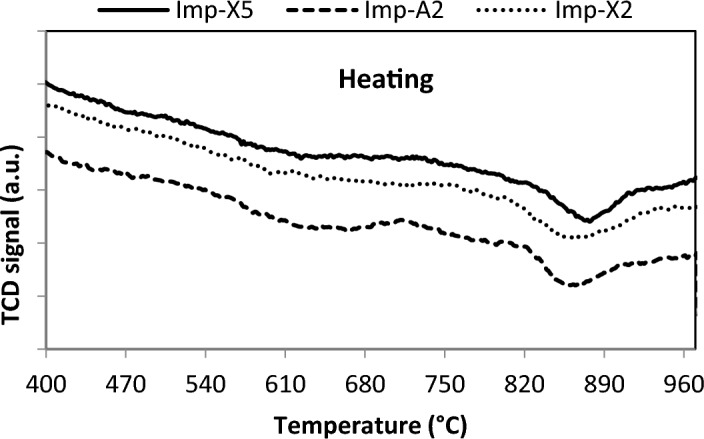
TPO profiles of catalysts during heating.

**Figure 5:**
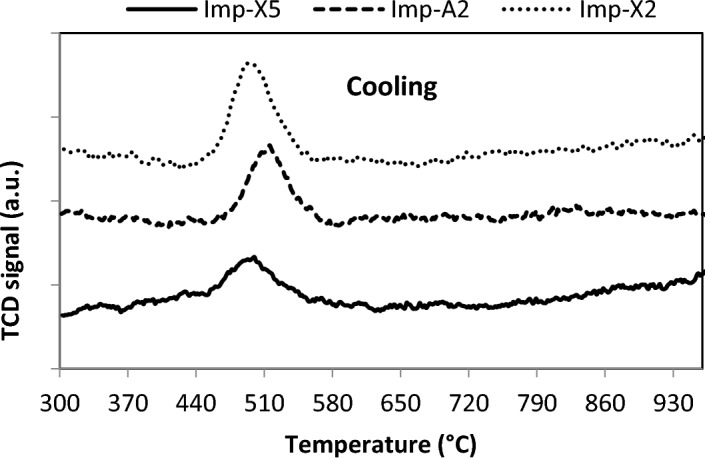
TPO profiles of catalysts during cooling.

In order to investigate the PdO-Pd^0^ transformation properties during raising and lowering the temperature, O_2_-TPO experiments were carried out. Figures 4 and 5 show the obtained results over Imp-Xy and Imp-A2 catalysts. The TPO profiles in all catalysts exhibit one negative peak representing oxygen desorption as the temperature is raised at about 850 °C (Fig. 4), and a positive peak representing oxygen consumption as the temperature is lowered from 1000 to 300 °C (Fig. 5). The negative peak is attributed to the decomposition of PdO species and the positive peak is assigned to the re-oxidation of metallic Pd species. The temperatures of PdO decomposition and its re-oxidation during raising and lowering the temperature shift to higher values for Zr-rich catalyst (Imp-X5) and aerogel derived catalyst (Imp-A2). This observation suggests that supercritical drying and/or increasing the Zr content improve the thermal stability of PdO. Similar results have been obtained by Zhou et al.^44^. In fact, they have found that in the presence of Ce and Zr oxides, the temperature of PdO decomposition and that of metallic Pd re-oxidation both shifted to higher values than the corresponding temperatures observed on Pd/Al_2_O_3_ catalysts.

#### Palladium reducibility

**Figure 6:**
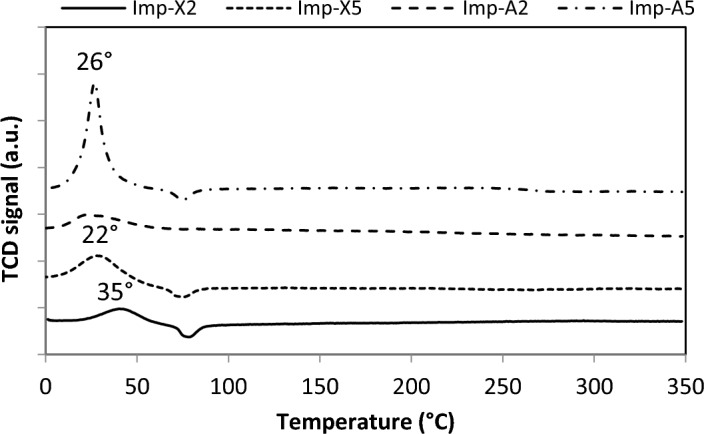
TPR profiles of the prepared catalysts.

The reducibility of supported palladium oxide is an important factor influencing its catalytic performance in methane combustion^45^. H_2_-temperature-programmed reduction was used to study the reducibility of the prepared catalysts (Fig. 6). For all samples investigated, there is a positive peak that is attributed to the reduction of PdO species dispersed on alumina-rich grains as reported by Yue et al.^46^. As can be seen, the catalyst Imp-X2 exhibits the positive peak at about 35 °C which is characteristic of the reduction of highly dispersed PdO species as mentioned by R. Zhou et al.^44^. This peak was shifted to lower temperature as the Zr content increases and/or the drying is done under supercritical conditions. This observation suggests that both Zr and supercritical drying increase the reducibility of PdO species. It also suggests a weak interaction between palladium species and the support in the samples Imp-A2, Imp-X5 and Imp-A5 and proves the good interaction between Pd and the support in Imp-X2 since the palladium reducibility temperature was shifted to relatively higher values. The negative peak detected at about 70 °C for Imp-Xy and Imp-A5 samples can be attributed to the decomposition of palladium hydride^47^.

#### Catalysts microstructure

**Figure 7:**
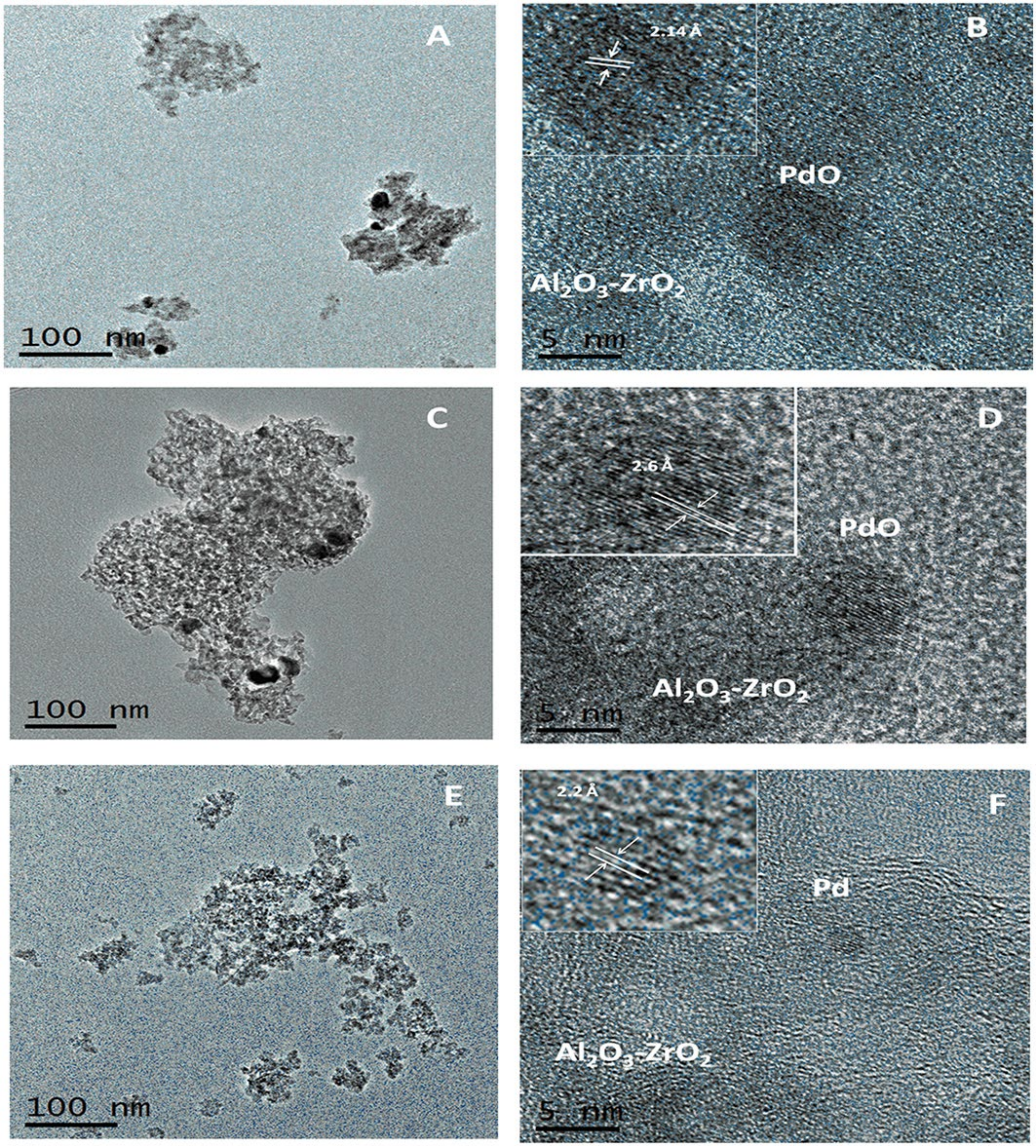
TEM and HRTEM images of the fresh catalysts: (**A**, **B**) Imp-X5, (**C**, **D**) Imp—X2, (**E**, **F**) Imp-A2.

TEM images of the samples Imp-X2, Imp-X5 and Imp-A2 confirm the porous nature of these catalysts (Fig. 7A,C,E). High-resolution TEM images show lattice fringes that allow to identify the nature of the nanoparticles. PdO particles were detected in xerogels catalysts. Figure 7B,D show nanoparticles with lattice fringes at 2.15 and 2.6 Å, which correspond to the (110) and (101) crystallographic planes of PdO, respectively^48–52^. These PdO nanoparticles measure about 5 nm in diameter. In contrast, the catalyst Imp-A2 contains much smaller palladium nanoparticles. They measure about 2–3 nm (Fig. 7F). These nanoparticles exhibit lattice fringes at 2.2 Å, which correspond to the (111) crystallographic planes of metallic Pd^48,53^. This result is in good accordance with those obtained after XRD analysis where we have noted the absence of peaks related to PdO in aerogels. However, that does not mean the inexistence of this oxide but maybe its particles are very small and/or well dispersed to not be detected. These two hypotheses could be proved by examining the TEM image and H_2_ chemisorption results of this sample. In fact, the dark spots observed on Imp-X5 and Imp-X2 samples are much bigger than those present in the TEM image of Imp-A2. Besides, for the same zirconium loading, palladium dispersion on aerogels is better. To conclude, we can say that all samples could contain both PdO and Pd particles but with predominance of PdO particles in xerogels and metallic palladium in aerogels. This hypothesis could be justified by XPS results discussed below. The predominance of metallic palladium in aerogels could be explained by the small size of PdO particles in comparison with those of metallic palladium, hence they will penetrate the large pores present in these samples and could not be detected.

### Catalysts surface composition

**Figure 8:**
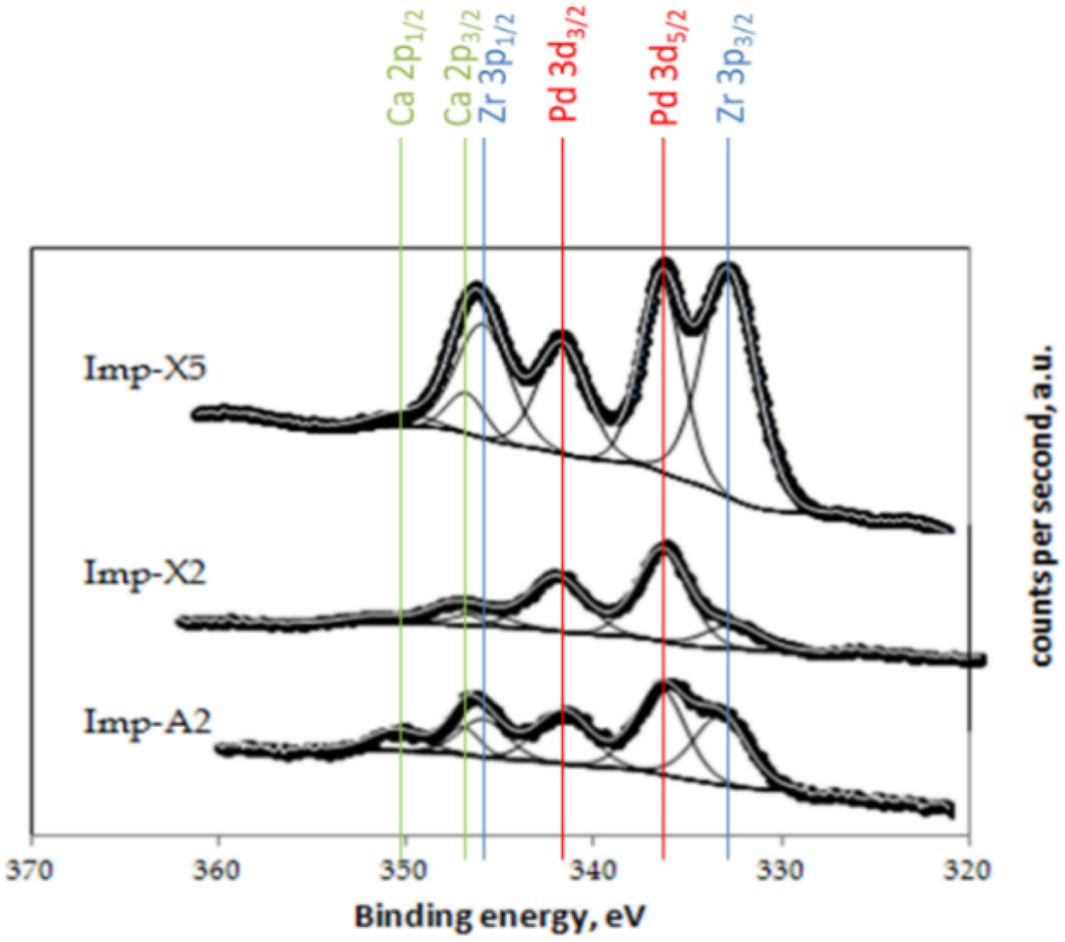
XP spectra and identification of the signals in the range of 360–320 eV.

X-ray photoelectron spectroscopy (XPS) was recorded over samples Imp-X2, Imp-A2 and Imp-X5. The corresponding Pd 3d spectra are shown in Fig. 8 and the corresponding binding energy values and surface atomic ratios are compiled in Table 3. The binding energies recorded for Pd 3d_5/2_ over all samples clearly indicate that palladium occurs as Pd(II) species (336.3–336.4 eV)^54–57^. The binding energies recorded for Pd 3d_3/2_ could be attributed for both Pd(II) and Pd(0) especially in the case of Imp-A2 since the corresponding peak is wide and it can be deconvoluted into at least 2 peaks: one relative to Pd(II) species at approximately 342 eV and the other attributed to Pd(0) species at 340 eV as advanced by C. Pan et al.^58^. On the other hand, the binding energy of zirconium 3d_5/2_ at 182.1–182.3 eV is consistent with ZrO_2_^57,59^ and that of aluminum 2p at 74.0–74.1 eV corresponds well to Al_2_O_3_, as expected^51,60^. However, important differences are observed when comparing the surface atomic ratios of Pd, Al and Zr (Table 4). The surface of the catalyst Imp-X5, which contains a higher amount of ZrO_2_, shows a strong enhancement of the Zr/Al ratio with respect to samples Imp-X2 and Imp-A2. This indicates that Zr is strongly segregated at the surface. Regarding the surface concentration of Pd, it is observed that the catalyst Imp-X2 contains a higher fraction of Pd exposed at the surface than the samples Imp-A2 and Imp-X5. Interestingly, the catalyst Imp-X2 is the one that performs better in the oxidation of methane. By comparing the XPS results with those obtained by H_2_ chemisorption, we note a contradiction specifically for the catalyst Imp-X2. In fact, this sample and based on H_2_ chemisorption exhibits the lowest palladium dispersion while the fraction of Pd on its surface is the highest based on XPS measurements. These incoherent results could be explained by the fact that the chemisorption stoichiometry of an adsorbate on metals could be changed if the metal surface is modified or by the formation of an alloy which could chemisorb a gas in a different manner than pure metal^61^. In our case, the two suggestions are possible and the alloy is probably formed between Pd and Al. The formation of the alloy could be consolidated by the good interaction between palladium and support proposed for the catalyst Imp-X2 due to the shift of the palladium reducibility temperature at higher values. Nevertheless, it will be interesting to confirm the formation of this alloy using a convenient technique.

**Table 3:** XPS binding energies of the fresh catalysts.

Catalyst	Pd 3d5/2 (eV)	Al 2p (eV)	Zr 3d5/2 (eV)
Imp—X2	336.3 ± 0.2	74.1 ± 0.2	182.3 ± 0.2
Imp—A2	336.4 ± 0.2	74.0 ± 0.2	182.3 ± 0.2
Imp—X5	336.4 ± 0.2	74.0 ± 0.2	182.1 ± 0.2

**Table 4:** XPS-derived atomic ratios of the fresh catalysts.

Catalyst	Surface atomic ratio (× 10^2^)		
	Pd/Al	Zr/Al	Pd/(Al + Zr)
Imp—X2	1.5	0.8	0.84
Imp—A2	0.4	0.7	0.24
Imp—X5	3.1	9.8	0.27

### Catalysts catalytic activity

**Figure 9:**
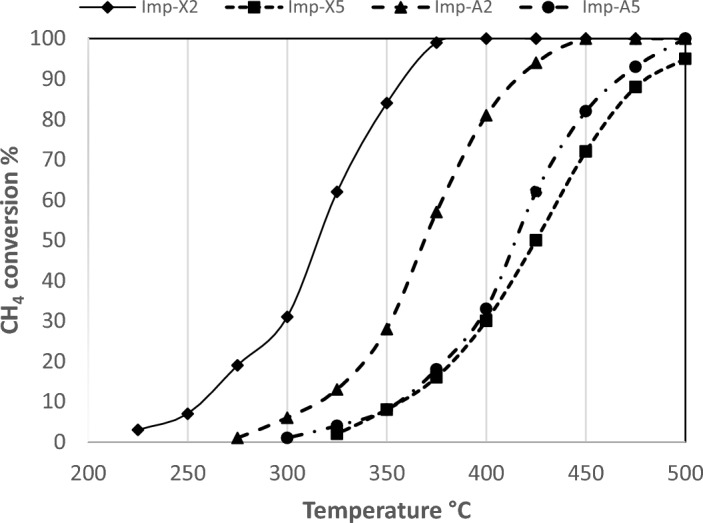
Catalytic behaviour of the prepared catalysts in methane oxidation.

The catalytic behaviour of the four investigated is shown in Fig. 9. All the tested catalysts exhibit a complete methane combustion at 500 °C. Differences in the catalytic activity were observed when decreasing temperature. At this stage, the better catalytic performance was observed on the catalyst Imp-X2 followed by the catalyst Imp-A2 while both aerogel and xerogel catalysts containing 5% of Zr exhibit lower and similar catalytic performances especially at temperatures ≤ 400 °C. The light-off temperature of Imp-X2, Imp-A2, Imp-A5 and Imp-X5 was 225 °C, 275 °C, 300 °C and 325 °C respectively. Differences in microstructure of all these samples is the principal cause of the big differences in the light off values. In fact, one of the factors explaining the catalytic behaviour differences observed on all samples is the moderate reducibility of PdO particles noted for the catalysts Imp-X2 and Imp-A2 compared to Imp-X5 and Imp-A5 samples. In fact, as mentioned before in a previous work^16^, catalysts with moderate reducibility and high oxygen exchange activity of PdO particles will have higher activity. Also, and based on the XPS study, a higher fraction of Pd was exposed at the surface of the catalyst Imp-X2^53^. So, this could be another factor explaining the best catalylic behaviour observed on this sample. Besides, the presence of highly dispersed PdO particles on this sample as deduced from the TPR characterization is another factor leading to the high catalytic performance^44^. The predominance of Pd(0) in aerogels could also be one of the reasons of their lower activities compared to xerogels. In fact, based on T.V. Choudhary et al.^62^, the methane combustion activity of the palladium catalysts is strongly influenced by the degree of PdO reduction and Pd oxidation, respectively; it decreases with increasing Pd^0^ content and increases with increasing PdO content. Stakheev et al.^63^ and Murata et al.^64^ found an increase in catalytic activity with increase in particle size and since Imp-X2 exhibits the highest Pd particles size (3.7 nm) based on chemisorption results and the best catalytic activity, this constatation could be retained in our case.

To conclude with our results, we can say that although the better dispersion and the smaller size of PdO particles observed on aerogels, these two factors have not contributed to the improvement of the catalytic activity of our system. This could be explained by the fact that the PdO sites accessible for chemisorption were not totally accessible as active sites for the surface reaction with methane and oxygen. This argument was advanced by D.M. Fertal et al.^65^ who have noted a decrease in the catalytic activity of one of their PdO/CeO_2_-γ Al_2_O_3_ samples despite the better palladium dispersion and the smaller PdO size. Besides, for both zirconium loadings (2 and 5%), xerogels are more active than aerogels with a great difference in the catalytic behaviour for zirconium content of 2%. The large pores obtained in Imp-A5 have made differences between xerogel and aerogel less pronounced. In fact, one of the possible contributions of the high porosity in this material is the improvement of effective diffusivity of reaction gases in the catalyst^41^.

By comparing the catalytic behaviour of the best performing sample (Imp-X2) with all our previous results^14–17^, much better methane conversion is obtained.

In a recent study done by J. Lin et al.^66^ based on the use of alumina modified with zirconia and ceria as support for xerogel palladium catalysts, they have found that reaction temperature for 90% methane conversion T90 is at about 390 °C for their best performing catalyst. By comparing this value with that of the best performing catalyst in our study (Imp-X2) estimated at about 360 °C, our result is better since T90 of our sample is lower and the loading of Zr needed to obtain this value is also lower (2 wt% vs. 5 wt%) . With a minimum quantity of Zr and without adding ceria, we succeeded to obtain a higher performing catalyst.

D.R. Fertal et al. have investigated palladium supported on alumina and ceria considered similar to our system^65^. High palladium and ceria percentages were used: 4% Pd and 20% Ce. Methane combustion is found to be complete (100%) in the temperature range: 275–450 °C which seems to be an excellent result. However, the low Pd and Zr loadings used in our case (0.5%Pd and 2% Zr) and their contribution in the catalytic performance of our system seems to be again better. Both metals are expensive and with just a minimum quantity of them, methane was sufficiently converted at low temperatures (84% of conversion at 350 °C).

## Conclusions

This study describes the effect of the drying mode on the properties of catalysts based on palladium and used for methane combustion. Improvement of the specific surface area and palladium dispersion was observed when drying under supercritical conditions while the best catalytic performance was obtained for the catalyst prepared with low zirconium content and dried under ordinary conditions. So, we can conclude that our objective is achieved since we have succeeded in the improvement of the catalytic activity of Pd/Al_2_O_3_-ZrO_2_ catalysts compared to our previous results. Other preparation parameters could be tested in future such as the use of solid exchange method. Also, other catalytic reactions could be performed by using the same catalytic system described in this study.

## Data availability

All data generated or analysed during this study are included in this published article [and its supplementary information files].
